# A systematic review of non-motor rTMS induced motor cortex plasticity

**DOI:** 10.3389/fnhum.2015.00416

**Published:** 2015-07-21

**Authors:** Grégory Nordmann, Valeriya Azorina, Berthold Langguth, Martin Schecklmann

**Affiliations:** ^1^Experimental and Clinical Neuroscience, University of RegensburgRegensburg, Germany; ^2^Department of Psychiatry and Psychotherapy, University of RegensburgRegensburg, Germany

**Keywords:** transcranial magnetic stimulation, TMS, cross-modal, plasticity, excitability, motor cortex

## Abstract

Motor cortex excitability can be measured by single- and paired-pulse transcranial magnetic stimulation (TMS). Repetitive transcranial magnetic stimulation (rTMS) can induce neuroplastic effects in stimulated and in functionally connected cortical regions. Due to its ability to non-invasively modulate cortical activity, rTMS has been investigated for the treatment of various neurological and psychiatric disorders. However, such studies revealed a high variability of both clinical and neuronal effects induced by rTMS. In order to better elucidate this meta-plasticity, rTMS-induced changes in motor cortex excitability have been monitored in various studies in a pre-post stimulation design. Here, we give a literature review of studies investigating motor cortex excitability changes as a neuronal marker for rTMS effects over non-motor cortical areas. A systematic literature review in April 2014 resulted in 29 articles in which motor cortex excitability was assessed before and after rTMS over non-motor areas. The majority of the studies focused on the stimulation of one of three separate cortical areas: the prefrontal area (17 studies), the cerebellum (8 studies), or the temporal cortex (3 studies). One study assessed the effects of multi-site rTMS. Most studies investigated healthy controls but some also stimulated patients with neuropsychiatric conditions (e.g., affective disorders, tinnitus). Methods and findings of the identified studies were highly variable showing no clear systematic pattern of interaction of non-motor rTMS with measures of motor cortex excitability. Based on the available literature, the measurement of motor cortex excitability changes before and after non-motor rTMS has only limited value in the investigation of rTMS related meta-plasticity as a neuronal state or as a trait marker for neuropsychiatric diseases. Our results do not suggest that there are systematic alterations of cortical excitability changes during rTMS treatment, which calls into question the practice of re-adjusting the stimulation intensity according to the motor threshold over the course of the treatment.

## Introduction

Repetitive transcranial magnetic stimulation (rTMS) is capable of modulating cortical excitability in a frequency dependent manner. High frequency rTMS (≥5 Hz) has been shown to induce long-term potentiation-like effects, whereas low frequency rTMS (≤1 Hz) typically leads to long-term depression like effects (Fitzgerald et al., 2006; Thut and Pascual-Leone, 2010). Facilitatory and inhibitory effects can also be induced by intermittent or continuous application of triplets of pulses with a frequency of 5 Hz [intermittent (iTBS) or continuous theta burst stimulation (cTBS)] (Huang et al., 2005). Basic mechanisms of different rTMS protocols over the motor cortex have been intensely evaluated. In these studies, motor cortex excitability has been measured before and after rTMS using electromyographic activity which has been recorded after single and paired pulses of TMS over the corresponding area of the motor homunculus. Typical measures are resting motor threshold (RMT), motor-evoked potentials (MEPs), short-interval cortical inhibition (SICI), intracortical facilitation (ICF), and cortical silent period (CSP).

Changes in cortical excitability pre vs. post rTMS are interpreted as measures of rTMS-induced changes in synaptic plasticity (Fitzgerald et al., 2006). In addition, motor cortex excitability measures have been investigated as potential markers for neuropsychiatric disease related factors (Radhu et al., 2013; Bunse et al., 2014). Both cross-sectional and longitudinal studies have been performed to evaluate motor cortex excitability as a potential trait-like and state-like parameter (Frank et al., 2014; Strube et al., 2014). In addition, the effect of specific pharmacologic or brain stimulation interventions on motor cortex excitability has been assessed in patients and controls and interpreted as evidence for disease-related alterations of neuroplasticity. For example, alterations of SICI and CSP have been reported in patients with schizophrenia (Daskalakis et al., 2002; Hasan et al., 2013). Since both SICI and CSP are mediated by inhibitory gamma-aminobutyric acid (GABA-ergic) interneurons within the primary motor cortex (Ziemann, 2004; Di Lazzaro et al., 2006; Ziemann et al., 2006), these findings have been interpreted as an indication for impaired GABA-mediated function in schizophrenia.

However, schizophrenic patients do not only differ from healthy controls in their motor cortex excitability, but also in their neuroplastic response to rTMS. Motor cortex excitability remained unchanged in schizophrenic patients after 1 Hz rTMS, whereas the same stimulation pattern reduced MEPs in healthy controls (Fitzgerald et al., 2004). Such meta-plastic alterations were not observed when the modulatory effect of neuroleptic treatment on motor cortex excitability was investigated. Additionally, repeated intake of quetiapine resulted in an increase of the CSP in both patients (Frank et al., 2014) and controls (Langguth et al., 2008). Abnormal motor cortex excitability and meta-plasticity in neuropsychiatric disorders might therefore reflect a general neural alteration in neurotransmitter activity in these disorders.

Moreover, it has been demonstrated by neuroimaging methods that rTMS-induced activity changes are not restricted to the directly stimulated area, but also occur in functionally connected remote areas (Paus et al., 2001; Strafella et al., 2001). For example, stimulation of the dorso-lateral prefrontal cortex is associated with blood flow changes in the anterior cingulate cortex (Paus et al., 2001; Esslinger et al., 2014) and dopamine release in the caudate nucleus (Strafella et al., 2001). Such cross-modal plasticity-like effects may be transferred via direct cortico-cortical connections, indirectly via multi-sensory association areas, or via subcortical interplay at the thalamic level as indicated by findings in synaesthesia and sensory deprivation (Bavelier and Neville, 2002; Leon-Sarmiento et al., 2005; Dovern et al., 2012; Rothen and Terhune, 2012). Based on these cross-modal interactions, measurements of motor cortex excitability have also been investigated as a potential neural marker for the effect of rTMS of non-motor areas, e.g., the dorsolateral prefrontal cortex or the temporal cortex. The interpretation of the results is challenging, since they are influenced by stimulation-related parameters (e.g., frequency, intensity, number of stimuli), disease-related meta-plastic alterations, and by structural and functional interactions between the stimulated area and the motor cortex which may in turn be altered in specific diseases.

In this literature review, we systematically analyzed studies that investigated the effects of non-motor rTMS on motor cortex plasticity. Our main questions were whether this approach is effective for: (i) measuring rTMS treatment effects in neuropsychiatric disorders; (ii) scrutinizing cross-modal plasticity between motor and non-motor areas in both the healthy and diseased brain.

## Materials and methods

A systematic literature search was performed in April 2014 using the databases Medline, PsycInfo and ScienceDirect. The full list of search terms related to rTMS and neuropsychiatric diseases was conducted using the Medline and PsycInfo thesauri as well as TMS-related literature. All relevant search terms and the search fields title (TIT), abstract (ABS), and keyword (KW) coincided across all three databases. Medline and PsycInfo were searched simultaneously through the EBSCO interface (reference). The search resulted in the total of *n* = 6473 sources (465 ScienceDirect, 6008 Medline and PsycInfo combined). The search strategy, including the combination of the search terms, is outlined in Table 1.

**Table 1:** **Search strategy for identification of relevant publications**.

**Databases: PsycInfo, Medline, ScienceDirect**		
AND	TIT ABS KW	rTMS OR repetitive transcranial magnetic stimulation OR repetitive trans-cranial magnetic stimulation OR TMS OR transcranial magnetic stimulation OR trans-cranial magnetic stimulation OR theta-burst stimulation OR theta burst stimulation OR TBS OR paired associative stimulation OR PAS
	TIT ABS KW	Excitability OR “cort^*^ excitability” OR “motor cort^*^ excitability” OR MT OR motor threshold OR “cortic^*^ threshold” OR rMT OR resting motor threshold OR resting-motor threshold OR MEP OR motor evoked potential^*^ OR motor-evoked potential^*^ OR double pulse OR double-pulse OR paired pulse OR paired-pulse OR single-pulse OR single pulse OR CSP OR “^*^ silent period^*^” OR SPD OR SICI OR short interval cortical inhibition OR “^*^cortical inhibition” OR “^*^cortical facilitation” OR ICF OR ^*^callosal inhibition OR ^*^callosal facilitation OR ^*^hemispheric inhibition OR ^*^hemispheric facilitation OR “TMS paradigm^*^” OR inter-threshold difference OR inter threshold difference OR ITD OR “D-wave^*^” OR “M-wave^*^” OR “I-wave^*^” OR “I wave^*^” OR “M wave^*^” OR “D wave”
Result (21.04.2014): *n* = 6473		

TIT, Title; ABS, Abstract; KW, Keyword. Understandable in the contex; for literature research

* *stands for a placeholder*.

After removal of duplicates, the number of sources was reduced to *n* = 6403. Titles and abstracts of all sources were assessed and a total of *n* = 6331 sources were determined incompatible and excluded (e.g., animal studies, reviews, and irrelevant topics). The 72 remaining papers were assessed in full length by the two authors VA and GN independently according to the inclusion/exclusion criteria listed below.

The selected studies had to include: (i) original data; (ii) repetitive TMS or theta-burst stimulation; (iii) stimulation of non-motor areas; (iv) pre-post motor cortex excitability measurements. Studies were excluded if: (i) ipsi- and contralateral motor or near motor areas (premotor cortex, supplementary motor cortex, primary motor cortex or somatosensory cortex) were stimulated (studies investigating cerebellar stimulation were included); (ii) non-repetitive TMS was used; (iii) paired-pulse stimulation was used alternating between non-motor and motor areas; (iv) other types of stimulation were used (transcranial direct current stimulation, deep brain stimulation etc.); (v) no pre-post changes in motor cortex excitability were reported; (vi) data were based on single data (case reports or case series if the sample size was below five). Although effects of stimulation of near-motor areas such as the premotor cortex (Buhmann et al., 2004) or of contralateral motor areas to measure interhemispheric influence (Plewnia et al., 2003) might be interesting it falls beyond the scope of this review.

## Results

### Overview of literature research

**Figure 1:**
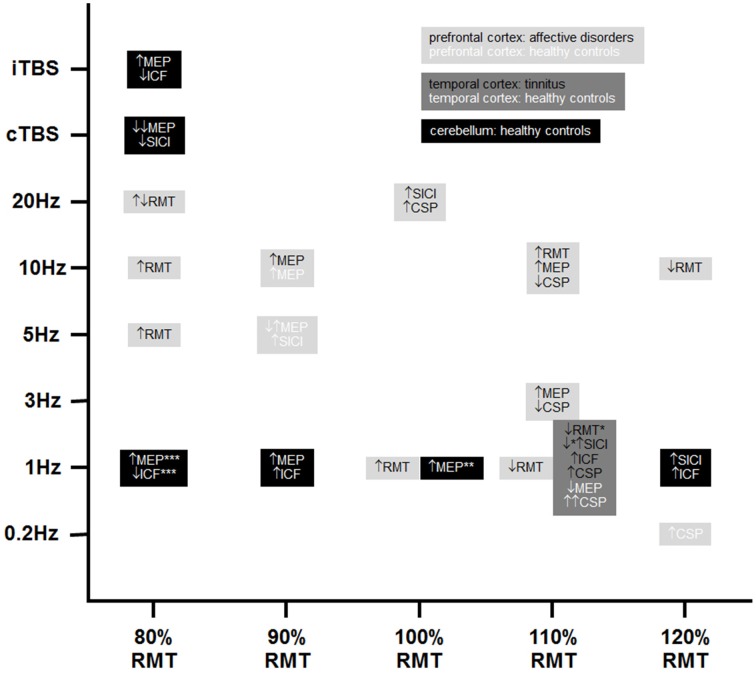
**Positive findings of TMS-induced changes in non-motor areas with respect to motor cortex excitability**. Please note that black, dark, and light gray represent different stimulation sites and white and black fonts represent healthy controls and patient groups, respectively. ↑, increases; ↔, no changes; ↓, decreases; CSP, cortical silent period; ICF, intracortical facilitation; MEP, motor evoked potential; RMT, resting motor threshold; SICI, short-interval intracortical inhibition; cTBS, continuous theta burst stimulation; iTBS, intermittent theta burst stimulation (8 s inter-burst interval); ^*^22 out of 116 patients received combined frontal and temporal stimulation; ^**^40% stimulator output instead of 100% RMT as stimulation intensity; ^***^90% active motor threshold over the inion instead of 80% RMT. Please note that increases in SICI means increases in inhibition and concomitantly a numeric decrease in the raw data.

The search strategy yielded 29 articles in the publication period from 1999 to 2014. Methods and respective results of these studies are listed in Tables 2A–**C**. A summary of positive results is provided in Figure 1.

**Table 2A:** **Overview of study parameters of prefrontal cortex rTMS studies investigating motor cortex excitability**.

**Authors**	**Sample (drop-offs excluded)**	**Stimulation sessions**	**Stimulation site**	**Treatment**	**Daily pulses**	**Findings**
Furukawa et al., 2010	10 HC 7 HC	1	Bilateral DLPFC sham	0.2 Hz, 120% RMT	100 100	↑ CSP in the verum condition ↔ RMT and MEP
Rollnik et al., 2000	18 HC	1	Left DLPFC Left OC (sham)	5 Hz, 90% RMT 5 Hz, 90 % RMT	60 60	↓ MEP in the verum group
Fierro et al., 2010	7 HC	1	Left DLPFC	5 Hz, 90% RMT	1800	↑ MEP, SICI after pain-induced decrease ↔ ICF
Grunhaus et al., 2003	19 MD 13 HC	1	Left DLPFC	10 Hz, 90 % RMT	1200	↑ MEP in both groups
Dolberg et al., 2002	46 MD 13 HC	20 20	Left DLPFC Left DLPFC	10 Hz, 90% RMT 10 Hz, 90% RMT	1200 1200	↔ RMT
Pallanti et al., 2012	28 MD	15	Right DLPFC	1 Hz, 110% RMT	420	↓ left RMT in responders ↔ right RMT
Nahas et al., 2003	11 BD 12 BD	10 10	Left DLPFC	5 Hz, 110% RMT sham	1600 1600	↔ RMT
Chistyakov et al., 2005	11 MD 6 MD 12 MD 6 MD 15 MD	10	Left DLPFC Right DLPFC Left DLPFC Right DLPFC sham	3 Hz, 110% RMT 3 Hz, 110% RMT 10 Hz, 100% RMT 10 Hz, 100% RMT Clomipramine	450 450 500 500	↑ RMT in the 10 Hz group ↑ MEP and ↓ CSP in responders in both left groups
Zarkowski et al., 2009	50 MD	15	Left DLPFC	10 Hz, 120% RMT sham	3000 3000	↔ RMT in both conditions; identification of sub-groups with low and high variability of RMT
Pretalli et al., 2012	75 UD+BD	10	Left DLPFC	10 Hz, 95% RMT	1200	↔ RMT identification of sub-groups with ↑, ↔, ↓ RMT independent from response
Croarkin et al., 2012	7 MD	25	Left DLPFC	10 Hz, 120% RMT	3000	↓ RMT
Spampinato et al., 2013	12 MD 10 MD	20	Left DLPFC sham	10 Hz, 120% RMT	3000	↔ RMT, SICI, ICF, CSP
Shajahan et al., 2002	5 MD 5 MD 5 MD	10	Left DLPFC	5 Hz, 80% RMT 10 Hz, 80% RMT 20 Hz, 80% RMT	500	↑ RMT during first week, followed by ↓ for all study arms
Triggs et al., 1999	9 MD	10	Left DLPFC	20 Hz, 80% RMT	2000	↓ RMT
Bajbouj et al., 2005	30 MD	10	Left DLPFC	20 Hz, 100% RMT	2000	↑ SICI, CSP in responders ↔ RMT, ICF
Mantovani et al., 2007	6 MD+PAD	10	Right DLPFC	1 Hz, 100% RMT	1200	↑ Right RMT
Ahmed et al., 2012	15 AD 15 AD 15 AD	5 5 5	Bilateral DLPFC Bilateral DLPFC sham	20 Hz, 90% RMT 1 Hz, 100% RMT	2000 2000 2000	↔ RMT

*Studies were listed according to specific samples and within the groups to stimulation frequency*.

*BD, bipolar disorder; HC, healthy controls; MD, major depression; PAD, panic disorder; UD, unipolar disorder; DLPFC, dorsolateral prefrontal cortex; OC, occipital cortex; ↑, increases; ↔, no changes; ↓, decreases; CSP, cortical silent period; ICF, intracortical facilitation; MEP, motor evoked potential; RMT, resting motor threshold; SICI, short-interval intracortical inhibition; please note that increases in SICI means increases in inhibition and concomitantly a numeric decrease in the raw data*.

The target regions of stimulation were the prefrontal cortex (PFC) (17 studies, see Table 2A), the temporal cortex (TC) (3 studies, see Table 2B), one combined stimulation of prefrontal and temporal regions, and the cerebellum (CRB) (8 studies, see Table 2C). The majority of the studies investigated either healthy controls (11 studies) or patient populations: affective disorders (bipolar disorder; major depression; panic disorder; uni-polar depression) (11 studies); tinnitus (2 studies); Alzheimer's (1 study); progressive supranuclear palsy (1 study). Three studies investigated both healthy controls and patients with a neuropsychiatric disorder: major depression (2 studies); Alzeihmers disease (1 study). Stimulation sessions varied between one and 25 days, daily pulses between 60 and 4000. All studies used either rTMS protocols with frequencies between 0.2 and 25 Hz or TBS (intermittent or continuous).

**Table 2B:** **Overview of study parameters of temporal cortex rTMS studies investigating motor cortex excitability**.

**Authors**	**Sample (drop-offs excluded)**	**Stimulation sessions**	**Stimulation site**	**Treatment**	**Daily pulses**	**Findings**
Eichhammer et al., 2007	17 HC 14 HC	5 5	Left STG sham	1 Hz, 110% RMT	2000 2000	↑ CSP in the verum group ↔ RMT, SICI, ICF
Lee et al., 2013	21 HC 5 HC	5	Right MTG sham	1 Hz, 110% RMT	1800	↑ MEP, ↓ CSP in the verum group ↔ RMT, SICI, and ICF
Langguth et al., 2007	10 TI	5	STG	1 Hz, 110% RMT sham	2000	↑ SICI, ICF, CSP associated with treatment response in the verum condition
Schecklmann et al., 2014	68 TI 26 TI 22 TI	10	AC AC Left DLPFC+ AC	1 Hz, 110% RMT 1 Hz, 110% RMT 20 Hz+1 Hz, 110% RMT	2000 4000 2000+2000	↑ RMT for all subjects ↓ SICI in responders ↔ ICF, CSP

*Studies were listed according to specific samples and within the groups to stimulation frequency*.

*HC, healthy controls; TI, tinnitus; AC, auditory cortex; DLPFC, dorsolateral prefrontal cortex; MTG, middle temporal gyrus; STG, superior temporal gyrus; ↑, increases; ↔, no changes; ↓, decreases; CSP, cortical silent period; ICF, intracortical facilitation; MEP, motor evoked potential; RMT, resting motor threshold; SICI, short-interval intracortical inhibition; please note that increases in SICI means increases in inhibition and concomitantly a numeric decrease in the raw data*.

**Table 2C:** **Overview of study parameters of cerebellum rTMS studies investigating motor cortex excitability**.

**Authors**	**Sample (drop-offs excluded)**	**Stimulation sessions**	**Stimulation site**	**Treatment**	**Daily pulses**	**Findings**
Gerschlager et al., 2002	8 HC 5 HC	1	Right CRB neck (control)	1 Hz, 40% SO	500	↑ MEP for CRB and neck stimulation
Oliveri et al., 2005	10 HC	1	Left CRB	1 Hz, 90% RMT	600	↑ MEP, ICF ↔ CSP
Fierro et al., 2007	8 HC	1	Right CRB neck (control)	1 Hz, 90% AMT at the inion	900	↓ ICF, ↑ MEP ↔ SICI
Langguth et al., 2008	10 HC	1	Medial CRB Right CRB Medial CRB Right CRB	1 Hz, 120% RMT 1 Hz, 120% RMT 10 Hz, 120% RMT 10 Hz, 120% RMT	1000	↑ SICI, ↓ ICF, ↔ RMT after 1 Hz ↔ RMT, SICI, ICF after 10 Hz
Di Lorenzo et al., 2013	12 AD 12 HC	1	Right CRB Right OC (control)	cTBS 80% AMT	600	↔ SICI, ICF
Koch et al., 2008	10/12 HC	1	Left CRB	iTBS, cTBS 80,90% AMT	600	↓ MEP, SICI after cTBS ↑ MEP, ↓ ICF after iTBS ↔ RMT
Li Voti et al., 2014	12 HC	1	Right CRB	cTBS 80% AMT	600	↓ MEP
Brusa et al., 2014	10 PSP	10	Left and right CRB	iTBS 80% AMT	1200	↔ RMT, SICI, ICF

*Studies were listed according to specific samples and within the groups to stimulation frequency*.

*AD, Alzheimer's disease; HC, healthy controls; PSP, Progressive Supranuclear Palsy; CRB, cerebellum; SO: stimulator output; ↑, increases; ↔, no changes; ↓, decreases; CSP, cortical silent period; ICF, intracortical facilitation; MEP, motor evoked potential; RMT, resting motor threshold; SICI, short-interval intracortical inhibition; cTBS, continuous theta burst stimulation; iTBS, intermittent theta burst stimulation (8s inter-burst interval); please note that increases in SICI means increases in inhibition and concomitantly a numeric decrease in the raw data*.

The effect of rTMS/TBS was investigated using many different indicators of efficacy: RMT (19 studies); MEP (11 Studies); SICI (12 studies); ICF (13 studies); CSP (9 studies). Inter-hemispheric measures, as well as changes in active motor threshold, cerebellar brain inhibition, F-waves, long-interval intra-cortical inhibition, and short-latency afferent inhibition are not reported in the following due to infrequent use (≤2). Because of the more direct connectivity between CRB and motor cortex in contrast to TC or PFC, results were reported separately for cerebellar and non-cerebellar stimulation. All studies with frontal or temporal stimulation used rTMS protocols. Four of the cerebellar stimulations used rTMS and four used TBS protocols.

In summary, studies showed high heterogeneity of study design. Furthermore, only seven out of these 29 articles used a control/sham condition and only four had a sample size over 30. Most of the studies analyzed the cross-modal plasticity effects in an explorative or *post-hoc* manner.

### RMT

Seventeen studies investigated the effect of frontal (14) or temporal (3) rTMS on changes in RMT. Two studies in patients with affective disorders reported increase of RMT (decreased excitability) after applying pulses to the dorsolateral PFC (DLPFC): one used 1200 pulses with 1 Hz over 10 days (Mantovani et al., 2007), the other used 500 pulses with 10 Hz over 10 days (Chistyakov et al., 2005). Another study in patients with depression described a time-dependent, frequency-independent (5, 10, and 20 Hz) inverted u-shaped characteristic of RMT development (Shajahan et al., 2002). Over the course of the 10-day treatment with 500 daily pulses over the DLPFC, they found an increase in RMT during the first 7 days followed by a decrease in the following 4 days. In contrast, three studies (two stimulating the DLPFC using 10/20 Hz in patients with major depression; one stimulating both the DLPFC with 20 Hz and the TC with 1 Hz in tinnitus patients) using at least 2000 pulses over at least 2 weeks described a decrease of RMT (Triggs et al., 1999; Croarkin et al., 2012; Schecklmann et al., 2014). However, in one of these studies the effect of a mean decrease of 1% stimulator output was small and only near significant due to the large sample size (Schecklmann et al., 2014). Additionally, RMT decreases after DLPFC stimulation were found in patients with affective disorders (1 Hz, 15 days, 420 daily pulses) and were associated with clinical response (Pallanti et al., 2012). The majority of the studies (10 in total: 8 DLPFC; 2 TC) did not reveal any changes in RMT independent of the treatment frequency, number of daily pulses, and treatment days, e.g., (Dolberg et al., 2002; Nahas et al., 2003; Ahmed et al., 2012). Within these 10 studies, sub-group-dependent changes of RMT (Pretalli et al., 2012) and sub-groups with low and high variability in RMT (Zarkowski et al., 2009) were reported.

For cerebellar stimulation, three studies investigated the effects of different TMS protocols (rTMS and TBS) on RMT values. No changes were reported (Langguth et al., 2008; Brusa et al., 2014).

### MEP

In six studies modulation of cortical excitability after frontal (5) and temporal (1) stimulation was investigated by measuring MEPs. Three studies found increased MEP values: one found an increase in MEP amplitude up to 30 min after rTMS stimulation of the DLPFC in both patients with major depression and control groups in a single-day study with 1200 pulses at 10 Hz (Grunhaus et al., 2003); the second study showed increased MEP amplitude only in major depression patients with marked clinical improvements after 10 stimulation sessions of the left DLPFC with either 3 or 10 Hz rTMS (Chistyakov et al., 2005); the third found that the effects of capsaicin-induced pain on MEP (decrease of MEP) could be reverted by acute 5 Hz rTMS with 1800 pulses applied in one session (Fierro et al., 2010). Decreased MEP amplitudes were reported in two studies with healthy controls stimulated with 5 Hz (1-day treatment with 60 pulses over the DLPFC) and 1 Hz pulse frequency (5-day treatment with 1800 daily pulses over the TC), respectively (Rollnik et al., 2000; Lee et al., 2013). One study consisting of one single session of 100 pulses at 0.2 Hz did not find an effect on MEP values (Furukawa et al., 2010).

Effects of cerebellar stimulation on MEPs were investigated in three studies using rTMS and in two studies with TBS. In two single-session studies with 500 and 600 pulses, respectively, MEPs were found to be increased after 1 Hz rTMS in healthy controls (Gerschlager et al., 2002; Oliveri et al., 2005). Additionally, one protocol with 900 pulses in one session applied with 1 Hz lead to progressively increasing MEP amplitudes (Fierro et al., 2007). Regarding TBS, one study found decreases and increases in MEP amplitude depending on the mode of TBS treatment applied to the lateral CRB (Koch et al., 2008): intermittent TBS (iTBS) lead to increases in MEP values, continuous TBS (cTBS) reduced MEP levels. Decreased MEP amplitudes were also reported from one iTBS study applied to the CRB (Li Voti et al., 2014). All TBS studies used 600 daily pulses.

### SICI

In seven studies, paired-pulse MEP measurements were used to assess changes in SICI after frontal (3) or temporal (4) cortical stimulation. Increased inhibition (decrease of the absolute value; decreased inhibition corresponds to an increase of the SICI value) after rTMS treatment over the DLPFC was reported in two studies: one investigated clinically responding major depression patients (ten treatment days, 2000 daily pulses, 20 Hz stimulation frequency) (Bajbouj et al., 2005) and the other healthy controls with capsaicin-induced acute pain (one session, 1800 pulses, 5 Hz stimulation frequency) (Fierro et al., 2010). Furthermore, a clinical study with tinnitus patients correlated rTMS-induced (5 treatment days, 2000 pulses, 1 Hz stimulation frequency) increases in SICI with reduction in tinnitus questionnaire scores (Langguth et al., 2007). The same authors reported inverse effects in a later retrospective analysis of a larger sample, i.e., decrease in SICI in responding tinnitus patients over the course of the trial (Schecklmann et al., 2014). Three studies with major depressive patients and healthy controls report no effects of rTMS on SICI values (Eichhammer et al., 2007; Lee et al., 2013; Spampinato et al., 2013).

For cerebellar stimulation, one study showed frequency-dependent alterations: while SICI values increased after 1 Hz stimulation, they remained unchanged after 10 Hz treatment applied in one single session with 1000 pulses (Langguth et al., 2008). Additionally, one study reported decreases in SICI after cTBS treatment (one session, 600 pulses) over the CRB. Four studies reported unchanged SICI values in response to: cTBS (one session, 600 pulses) (Grunhaus et al., 2003); iTBS treatment (one session, 1200 pulses), both in patients with neurological diseases (Fierro et al., 2007; Koch et al., 2008); one RMT study in healthy controls (Di Lorenzo et al., 2013).

### ICF

Regarding ICF, increases were reported after low-frequency stimulation over the TC in tinnitus patients who clinically responded to rTMS treatment and after stimulation over the CRB in healthy controls (Oliveri et al., 2005; Langguth et al., 2007). Another CRB study, performed in healthy controls, showed increasing effects on ICF after low-frequency stimulation (1 Hz), whereas ICF remained unchanged after high-frequency stimulation (10 Hz) (Langguth et al., 2008). In two other studies stimulation with 1 Hz rTMS and iTBS resulted in decreased ICF (Fierro et al., 2007; Koch et al., 2008). Eight studies (three frontal, three temporal, two cerebellar) found no effect on ICF.

### CSP

Eight studies investigated the effect of rTMS on CSP duration after frontal (4) or temporal (4) stimulation. Five studies reported increased CSP after rTMS treatment; three (1 DLPFC, 2 TC) using low-frequency protocols for 1 or 5 days in healthy controls (Eichhammer et al., 2007; Furukawa et al., 2010; Lee et al., 2013); one in major depression (20 Hz, DLPFC) (Bajbouj et al., 2005); one in tinnitus patients (1 Hz, TC) (Langguth et al., 2007). In the patient studies, an increase of the CSP was only detected in clinically responding patients. Association of CSP and treatment response could not be replicated in a later retrospective analysis of a larger sample of tinnitus patients (Schecklmann et al., 2014). Reduction of CSP duration was reported only in one study in major depression patients who responded to 3 or 10 Hz with 450 or 500 daily pulses over 10 days (Chistyakov et al., 2005). One study in depression patients using DLPFC stimulation did not find changes in CSP (one session?, how many pulses?, 10 Hz stimulation frequency) (Spampinato et al., 2013). Additionally, one study reported no effect of cerebellar rTMS on CSP duration in healthy controls (one session, 600 pulses, low frequency stimulation) (Oliveri et al., 2005).

### Explorative analysis

In summary, all results and all study parameters showed high variability and no clear systematic pattern in our primary investigation (Tables 2A–C). On overview of increases, no changes, and increases of different measures of motor cortex excitability in dependence from stimulation site is given in Table 3 affirming the picture of no clear systematic pattern. Furthermore, the number of positive and of null findings can also be extracted from this table again showing no clear positive effects for one parameter. Next, we tried to reveal literature-inherent consistency for changes in motor cortex excitability that had been obscured by the heterogeneity of study designs by two approaches. First, we plotted only the significant findings with respect to the stimulated site, the investigated sample, the stimulation frequency, and the stimulation intensity (Figure 1). Second, we tried to identify pairs/groups of studies with similar study designs. We concentrated on studies in patients with affective disorders in which the effects of high-frequency rTMS over the DLPFC on the RMT were investigated, resulting in three paired studies and one group of three studies (Table 4). Among the three pairs, two showed concordant effects and one divergent effect. In the three matched studies, two showed convergent effects. The results of these nine studies indicate a dosage-dependent trend of rTMS effects on RMT values. Stimulation protocols with low dosage (as defined by low stimulation intensity, low number of treatment sessions, and low number of pulses/session) led to increases in RMT; no RMT changes were seen after rTMS at moderate dosage; decreased RMT was evident after rTMS at high dosage.

**Table 3:** **Number of different measures of motor cortex excitability with increases, no changes (null findings), and decreases with respect to the different stimulation sites**.

		**Resting motor threshold**	**Motor evoked potential**	**Short-interval intracortical inhibition**	**Intracortical facilitation**	**Cortical silent period**
Frontal	↑	3	3	2	0	2
	↔	9	1	1	3	1
	↓	3	1	0	0	1
Temporal	↑	0	0	1	1	3
	↔	2	0	2	3	1
	↓	1	1	1	0	0
Cerebellum	↑	0	4	1	2	0
	↔	4	0	4	3	1
	↓	0	2	1	2	0
Frontal + temporal + cerebellum (sum)	↑	3	7	4	3	5
	↔	15	1	7	9	3
	↓	4	4	2	2	1

*Please note that numbers are not consistent with numbers of studies as several studies used several stimulation protocols*.

*↑, increases; ↔, no changes; ↓, decreases over the course of the repetitive transcranial magnetic stimulation*.

**Table 4:** **Combinations of studies with similar study designs**.

**Authors**	**Sample size**	**Days treated**	**Stimulation frequency (Hz)**	**Stimulation intensity (% RMT)**	**Daily pulses**	**Findings for the RMT**
Shajahan et al., 2002	15	10	5, 10, 20	80	500	↑
Chistyakov et al., 2005	18	10	10	100	500	↑
Pretalli et al., 2012	75	10	10	95	1200	↔
Dolberg et al., 2002	46	20	10	90	1200	↔
Bajbouj et al., 2005	30	10	20	100	2000	↔
Triggs et al., 1999	9	10	20	80	2000	↓
Zarkowski et al., 2009	50	15	10	120	3000	↔
Croarkin et al., 2012	7	25	10	120	3000	↓
Spampinato et al., 2013	12	20	10	120	3000	↔

*All studies stimulated the left dorsolateral prefrontal cortex in only patients with affective disorders. Variable of interest was the resting motor threshold*.

*RMT, resting motor threshold; ↑, increases; ↔, no changes; ↓, decreases in RMT over the course of the repetitive transcranial magnetic stimulation*.

## Discussion

In this systematic review we report data from 29 studies, which were identified by systematic literature research, in which changes of motor cortex excitability induced by non-motor rTMS or TBS have been investigated.

In 19 of the 29 studies, the effect of different stimulation protocols on RMT was investigated. The results of these studies did not show any clear evidence for a systematic influence of non-motor rTMS on RMT. This is of high practical relevance, since stimulation intensity is typically adjusted to individual RMT. With no tendency toward a systematic modulation of the RMT during treatment, our results provide no further support for the recommendation to re-measure the RMT over the course of the treatment (Zarkowski et al., 2009) and to adjust the stimulation intensity for reasons of efficacy and safety. Decreases of RMT without adjustment of stimulation intensity might result in too high stimulation intensity, and increases of RMT without adjustment of treatment intensity may eventually lead to stimulation intensities below the effective dosage. We are aware that the lack of a systematic effect of non-motor stimulation on the RMT does not exclude possible effects in subgroups or transient effects. However, only with evidence from future studies that reveal stable and reliable RMT increases and decreases in a subgroup of patients, should regular measurement of the RMT and adjustment of stimulation intensity be recommended.

For MEPs and SICI, no systematic changes could be identified. The ICF does not seem to be sensitive to any kind of non-motor rTMS intervention, since the majority of the studies reported no changes for this parameter. For the CSP, five out of eight studies showed increases independent of any of the experimental parameters (frequency, sample, etc.). The CSP in known to be mediated by the GABA_B_ receptor (Werhahn et al., 1999), which is assumed to be involved in the aetiopathology of both affective disorders and tinnitus. Schizophrenia is also characterized by impaired inhibitory mechanisms as elicited by reduced motor cortex excitability (Hasan et al., 2013). Moreover, neuroleptic treatment has been shown to increase the CSP (Frank et al., 2014), suggesting that CSP changes might mirror plasticity-related state-like effects. Remarkably, apart from a case report in which reduction of auditory hallucinations after temporo-parietal low-frequency rTMS was reflected by an increase of the CSP (Langguth et al., 2006), there is no longitudinal study of motor cortex excitability changes during rTMS treatment of schizophrenic patients.

The observed high variability in the changes of the dependent variables might be related to the high heterogeneity of the investigated studies with respect to study parameters (samples, stimulation pattern, stimulation frequency, stimulation site, stimulation intensity, number of pulses, number of sessions). With such a high number of variables and since most studies differ in several variables, comparisons across studies are difficult and the interpretation of differences in the results is challenging. Also, although established, the parameters investigated here are not free from controversy: recently, a study systematically investigated the reliability of MEPs and found out that at least 30 repetitions are necessary for stable MEPs (Cuypers et al., 2014). This is in contrast with common practice, as can be seen in well referenced articles (Gerschlager et al., 2002; Schecklmann et al., 2014) and as suggested by textbooks (Siebner and Ziemann, 2007). In addition, further measurement methods (e.g., conditioning TMS pulse intensity and inter-stimulus intervals in the paired-pulse paradigms), for which we abstained from reporting them due to shortage of space, might also contribute to the high variability. As an example, 1 Hz rTMS over cerebellar cortex resulted in different effects on ICF in three different studies, which may be related to differently chosen inter-stimulus intervals in these studies (Oliveri et al., 2005; Fierro et al., 2007; Langguth et al., 2008). Furthermore, in general, the methodological quality should be increased by investigating bigger samples, by using control/sham conditions, and by using the topic of cross-modal plasticity not only as side hypothesis and additional *post-hoc* analysis.

We used two different approaches to systematically compare the available studies in spite of the mentioned difficulties. First, we classified the studies with respect to the stimulated cortical site (prefrontal cortex, temporal cortex, cerebellum) and the investigated sample (healthy vs. patient papulations) (see Tables 2A–C) and plotted only the significant findings with respect to the stimulated site, the investigated sample, the stimulation frequency, and the stimulation intensity. Despite remarkable variability in stimulation parameters for all three stimulation sites, we could detect certain relationships between stimulation sites and stimulation parameters. Studies investigating prefrontal stimulation were characterized by high variability with respect to stimulation frequency and intensity, while temporal cortex stimulation was exclusively performed at 1 Hz and 110% RMT stimulation intensity. The heterogeneity of cerebellar stimulation protocols was somewhere in between. Although the CRB has closer structural and functional connections to the motor cortex, clear patterns of cerebellar stimulation on motor cortex excitability are not detectable. All three tonic 1 Hz rTMS studies investigating MEPs showed increases, indicating facilitatory effects. Along with these effects, two further studies showed increases in ICF. However, two other studies showed decreases in ICF and increases in SICI, which are inhibitory. In the motor cortex, 1 Hz rTMS causes inhibition, leading to the speculation that cerebellar 1 Hz rTMS may result in disinhibition of the motor cortex. However, cerebellar TBS studies showed the opposite pattern: cTBS, which is supposed to have inhibitory effects on the stimulated area, reduced motor cortex excitability, and facilitatory iTBS of the cerebellum increased motor cortex excitability as shown by MEPs and decreased excitability as shown by ICF in one study. This might be a suggestion that neither the stimulated area, nor the technical parameters were exclusively the reason for the high variability found in the reported studies. Further, limiting factors were evident: (i) small sample sizes, data on tinnitus showed controversial results, i.e., increase in SICI in the responder group in a sample of 10 patients (Langguth et al., 2007) and a decrease in SICI in the responder group in a sample of 116 patients (Schecklmann et al., 2014); (ii) medication, pharmacological status is a potential confounder since anti-depressants, which are prescribed in affective disorders and also in tinnitus, interact with neurotransmitter systems involved in motor cortex plasticity; (iii) sham controls, only six studies included a sham treatment to control for unspecific effects over time.

Our second approach was to compare studies with similar study designs. Here again, high-frequency rTMS studies of DLPFC in affective disorders with RMT as a dependent variable showed no clear pattern upon first investigation (Table 4). Speculatively, a dose effect (defined as the combination of number of treated days, daily pulses, and stimulation intensity) can be seen with increases of RMT under low dosage, no changes under moderate dosage, and decreases under high dosage of rTMS treatment. This is in line with findings from TBS studies. They showed that a prolongation of TBS over the motor cortex can diminish or even reverse neuroplastic after-effects (Gamboa et al., 2010). However, these conclusions are highly speculative, since this has not been systematically investigated.

In conclusion, we could not find clear evidence for cross-modal motor cortex plasticity from rTMS applied to non-motor cortical areas. Both the methodological constraints of the available studies as well as the intrinsic variability of brain function within single cortical sites and networks may play a role for the lack of clear systematic effects. It is also known from rTMS studies in motor cortex that interindividual variability is high (Ridding and Ziemann, 2010). Even the well-known and often described inhibitory effect of low-frequency continuous rTMS is subject to heterogeneity (Thut and Pascual-Leone, 2010). Several factors influencing motor cortex excitability and plasticity have previously been identified, i.e., attention (Stefan et al., 2004), hormone status (Inghilleri et al., 2004), history of synaptic activity (Siebner et al., 2004; Weisz et al., 2012) and the ongoing activity (Schulz et al., 2014) of the stimulated cortical area. The investigation of motor excitability changes after stimulation of brain regions more directly involved in motor execution and preparation such as the inferior frontal cortex (response inhibition) and the parietal cortex (sensorimotor integration) in a paired pulse design might be better suited for the characterization of remote rTMS effects (Van Campen et al., 2013; Chao et al., 2015). Additionally, the combination of TMS with electroencephalography, near infrared spectroscopy or functional magnetic resonance imaging might be more useful to identify cross-modal interactions induced by rTMS. However, based on the heterogeneity and limited methodological quality of the studies, these suggestions are speculative and several future studies with higher methodological standards (bigger sample size, sham-controlled designs) are recommended. Nonetheless, we carefully reported relevant parameters which turned out to be associated with high variability. “Balanced scholarly reviews might be more appropriate to give an overview about the state of the field, and suggest future directions of research. This would, however, include a meaningful discussion of heterogeneous study results, which also takes into account presumably discernable physiological effects of experimental protocol differences.” (Antal et al., 2015) (see also for discussion Nitsche et al., 2015).

### Conflict of interest statement

The authors declare that the research was conducted in the absence of any commercial or financial relationships that could be construed as a potential conflict of interest.
